# Spatial and temporal parasite dynamics: microhabitat preferences and infection progression of two co-infecting gyrodactylids

**DOI:** 10.1186/s13071-022-05471-9

**Published:** 2022-09-24

**Authors:** Clement Twumasi, Owen Jones, Joanne Cable

**Affiliations:** 1 School of Mathematics, Cardiff University, Abacws, CF24 4AG Cardiff, UK; 2 School of Biosciences, Cardiff University, Sir Martin Evans Building, CF10 3AX Cardiff, UK; 3 Department of Statistics, Oxford University, 29 St Giles’, OX1 3LB Oxford, UK

**Keywords:** *Gyrodactylus turnbulli*, *Gyrodactylus bullatarudis*, Multi-state Markov Model, Survival probability, Infection progression, Parasite virulence

## Abstract

**Background:**

Mathematical modelling of host-parasite systems has seen tremendous developments and broad applications in theoretical and applied ecology. The current study focuses on the infection dynamics of a gyrodactylid-fish system. Previous experimental studies have explored the infrapopulation dynamics of co-infecting ectoparasites, *Gyrodactylus turnbulli* and *G. bullatarudis*, on their fish host, *Poecilia reticulata*, but questions remain about parasite microhabitat preferences, host survival and parasite virulence over time. Here, we use more advanced statistics and a sophisticated mathematical model to investigate these questions based on empirical data to add to our understanding of this gyrodactylid-fish system.

**Methods:**

A rank-based multivariate Kruskal-Wallis test coupled with its post-hoc tests and graphical summaries were used to investigate the spatial and temporal parasite distribution of different gyrodactylid strains across different host populations. By adapting a multi-state Markov model that extends the standard survival models, we improved previous estimates of survival probabilities. Finally, we quantified parasite virulence of three different strains as a function of host mortality and recovery across different fish stocks and sexes.

**Results:**

We confirmed that the captive-bred *G. turnbulli* and wild *G. bullatarudis* strains preferred the caudal and rostral regions respectively across different fish stocks; however, the wild *G. turnbulli* strain changed microhabitat preference over time, indicating microhabitat preference of gyrodactylids is host and time dependent. The average time of host infection before recovery or death was between 6 and 14 days. For this gyrodactylid-fish system, a longer period of host infection led to a higher chance of host recovery. Parasite-related mortalities are host, sex and time dependent, whereas fish size is confirmed to be the key determinant of host recovery.

**Conclusion:**

From existing empirical data, we provided new insights into the gyrodactylid-fish system. This study could inform the modelling of other host-parasite interactions where the entire infection history of the host is of interest by adapting multi-state Markov models. Such models are under-utilised in parasitological studies and could be expanded to estimate relevant epidemiological traits concerning parasite virulence and host survival.

**Graphical Abstract:**

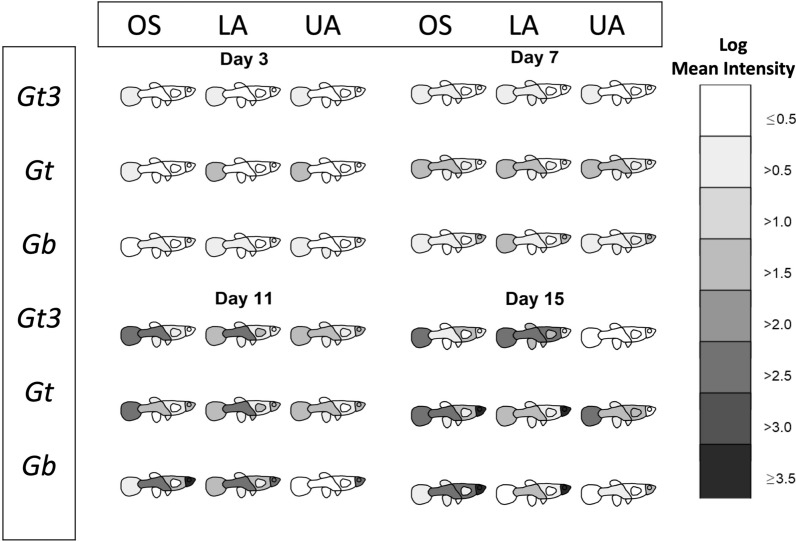

**Supplementary Information:**

The online version contains supplementary material available at 10.1186/s13071-022-05471-9.

## Background

In the field of theoretical and applied ecology, mathematical modelling of host-parasite systems has seen tremendous advancement and applicability [1–4]. Mathematical models provide a logical framework for developing, testing and evaluating ecological hypotheses and biological systems. These models can be categorised as individual-based models (IBMs), population-based models (PBMs) or hybrid models (i.e., integration of IBMs and PBMs) [5]. IBMs strictly model each individual by tracking the state of each member of the population, whereas PBMs track the total number of individuals in each state. Common modelling setbacks often lie with the models’ underlying assumptions being too simple or too complex [6]. For instance, many traditional ecological or epidemiological models (e.g., logistic, Lotka-Volterra predator-prey and compartmental models) assume that all individuals (within a subgroup) are identical and can be lumped together to represent the population size (as a single state variable) [7, 8]. This population lumpability means that all details regarding individual physiological and behavioural traits (determined by their specific genetic, age structure and other environmental factors) are lost. For spatially dependent systems, individuals typically affect other organisms within their spatial-temporal neighbourhood [9, 10]. Nevertheless, the data-generating processes that characterise parasite aggregation in the standard statistical frameworks are mostly not explicitly described or further explored [7]. Consequently, many studies have been conducted to bridge these modelling gaps associated with PBMs by adopting IBMs through computer simulations [11]. Incorporating evidence from the individual level to investigate processes at the population (e.g., during survival analysis), community and ecosystem levels can also improve PBMs (through hybrid models by leveraging the respective advantages of IBMs and PBMs) [12].

Through survival analysis, an individual’s infection history can be modelled as a two-stage process, with the simplest transition being from “alive” to “dead” state [13]. In such instances, we often adopt the standard logistic regression or Cox proportional-hazards regression to investigate risk factors of host mortality and to estimate hazard rates, while the non-parametric Kaplan-Meier method is used to estimate survival curves from censored data [14]. However, in most longitudinal studies, the “alive” state could further be divided into two or more intermediate (transient) phases, each corresponding to a different stage of an infection [15]. Multi-state models (MSMs) allow for time-to-event longitudinal data analysis in which surviving individuals may have different health outcomes over time. MSMs can be considered a type of hybrid model since they model several events in a given population while capturing population heterogeneity based on each individual’s infection history. A change of (infection or disease) state is considered a transition or an event. Estimating progression rates, transition probabilities, the mean sojourn time in a given state and analysing the effects of individual risk factors, survival rates and prognostic forecasting are all areas of interest under multi-state modelling [16]. For biomedical applications, clinical symptoms (such as bleeding episodes), biological markers (e.g. CD4 cell counts or serum immunoglobulin levels), scale of the disease (e.g. stages of cancer or HIV infection), or a non-fatal complication during infection progression (e.g. cancer recurrence) could all be used to classify states [16]. The states could be transient or absorbing if no other transitions could emerge from the state (e.g. after a death event). Particularly in medical settings, it is common that the exact time for some individuals to transition into an absorbing state (e.g. death) after the beginning of the study may be unknown or never quantified if occurring after the observation period. This leads to the issue of censoring in survival analysis (namely, right-censored, left-censored and interval-censored observational times), and the censoring effect must be included in multi-state modelling, especially when constructing the model’s likelihood function [16]. The complexity of an MSM in any time-to-event modelling problem depends on the number of states and all possible transitions. Hence, a more robust model (such as MSM) is useful to employ when studying host-parasite infection dynamics (including host survival and parasite virulence) to reflect the biological realism of the system under study and improve upon the estimation of risk parameters such as survival probabilities and hazard ratios [17].

Hence, multi-state models are considered a natural extension of the standard survival models [18, 19]. Andersen and Borgan [20] and Hoem et al. [21] previously reviewed Markov models, and Cox and Miller [22] broadly discussed multi-state models. In epidemiology, the states of the process could also be defined as disease outcomes such as healthy, exposed, infected, diseased with complications, or dead. The state structure (which is not unique) describes the states and the various transitions between them. For each possible transition, an MSM is specified entirely by its state structure (defined by the transition rate at which an event occurs over time) and the form of the hazard function for the respective transitions (given individuals’ characteristics or covariates). Moreover, an MSM can either be time homogeneous (if the transition intensities or rates of the Markov chain are invariant over time) or time inhomogeneous (in the case of time-varying transition rates). Also, the time-space can be either discrete or continuous. Other studies of infection diseases have employed discrete-time multi-state Markov models (e.g.,[23, 24]). Comprehensive and flexible software packages (e.g. *msm* and *msSurv* R packages) help modellers fit any proposed continuous-time multi-state Markov model (for a given biological system) based on panel or longitudinal data and well-defined transition intensities [25, 26].

In population demography, there are three broad areas for adopting MSMs [27]. The first approach is that this class of models can be used to generalise the basic life table to a more general nonhierarchical increment-decrement life table [28]. These linear models for homogeneous groups are developed using aggregate level data and are based on Markov chains. They are related to multi-state projection models, which result in asymptotic populations that are stable or temporarily stable. The second approach of MSMs (dubbed multi-state event-history models) employs event history analysis based on individual data to allow interactions between different processes and incorporate population heterogeneity [29]. This heterogeneity may be time dependent and vary according to origin or destination state. In the third approach (used for contextual and multilevel multiprocess models), both individual and aggregate measures are analysed simultaneously (in a hybrid manner). Contextual models are a straightforward extension of traditional modelling techniques, including aggregation, where the behaviours of individuals in the same context are considered independent [30]. Software like the *aML* package can handle fitting such models [31]. Multilevel multiprocess models are simultaneous equation systems that include multilevel hazard equations with correlated random effects (due to within-group dependence being introduced). One of the modern statistical softwares used for multilevel multiprocess modelling is *gsem* [32]. However, MSMs are rarely used for studying veterinary or wildlife host-parasite interactions. In the current study, for the first time, MSM is used to investigate the infection progression of two co-infecting gyrodactylids across different fish hosts.

Gyrodactylids are common fish parasites [33]. *Gyrodactylus salaris* alone caused epidemics among farmed salmonids, which resulted in death of up to 86% of salmon in infected rivers [34, 35]. They are monogenean ectoparasites that are ubiquitous on teleosts [36]. Amongst the well-studied Trinidadian guppy populations, gyrodactylids are the dominant parasites ($\ge 42\%$ prevalence, 3.3 mean intensity; [37]). The prevalence of *Gyrodactylus* species varies spatially across watercourses (lower, mid and upper courses of the rivers or lakes) and temporally among Trinidadian populations and between host sexes [38]. *Gyrodactylus* prevalence is higher in female guppies, but only in lower courses [38], predominately because of fish shoaling behaviour [39, 40]. The parasites have no specific transmission stage and transfer from fish to fish occurs during host contact. Their reproductive mode is similar to that of microparasites with replication occurring directly on the host (reviewed by [34]). Their hyperviviparous nature and short generation times ($<24$ h at $25^\circ \hbox {C}$; [41]) can cause population explosions with substantial spatial and temporal variation amongst different species (e.g. [42–46]). Many infect the skin and fins; others occur predominantly on the gills [44, 47]. *Gyrodactylus turnbulli* and *G. bullatarudis*, which both infect the guppy (*Poecilia reticulata*), niche partition with *G. turnbulli* occurring caudally [48] and *G. bullatarudis* rostrally [49]. According to Harris [48], as individual host infections with *G. turnbulli* progress, parasites migrate from the caudal fin and body to the pectoral, pelvic, dorsal and anal fins, a migration to potentially facilitate transmission. Gyrodactylids may also move to optimise feeding, reduce competition and avoid localised immune reactions [34, 50–54], the scorched earth hypothesis [46]. Although the respective caudal and rostral preferences of *G. turnbulli* and *G. bullatarudis* on the host are well reported (e.g. [48, 49]), consistency over time and across different fish stocks is not.

Host survival following gyrodactylid infection was previously explored by Cable and van Oosterhout [45]. They showed that mortality of guppies differed significantly between fish stocks for each parasite strain. From their experimental study, guppies were categorised according to whether they: (i) fought off the infection, (ii) remained infected, or (iii) died while infected. The fate of these guppies was predominantly affected by fish size, such that smaller guppies were more likely to clear the infection, while larger fish either died or remained infected beyond the end of the study period [45]. Parasites and their hosts compete for survival. Such co-evolutionary interactions drive virulence originating from parasite pathogenesis and host defence [55]. Together, measures of host mortality, host resistance, host recovery, mutation, superinfection, host heterogeneity and mode of transmission all contribute to explain parasite virulence [55]. Significant heterogeneity in virulence exists between *Gyrodactylus turnbulli* and *Gyrodactylus bullatarudis* strains [45] based on host mortality, host resistance and host heterogeneity, with less emphasis on host recovery as a measure of virulence [45, 56]. Although the proportion of gyrodactylid parasite-induced causalities on different fish host has been reported (e.g. [53]), the virulence of the three gyrodactylid strains on different fish stocks has not been quantified over time while accounting for possible changes in host infection status before host mortality may occur.

The current study focuses on the spatial and temporal infection dynamics of the gyrodactylid-fish system by providing new epidemiological insights with the help of a robust time-inhomogeneous MSM, and a rank-based multivariate Kruskal-Wallis test coupled with its post-hoc tests. The time-inhomogeneous MSM is considered to analyse longitudinal survival data (instead of its time-homogeneous version) since transition intensities may naturally differ across individuals or time-varying covariates. We examine gyrodactylid microhabitat preference of three parasite strains (two strains of *Gyrodactylus turnbulli* and one strain of *G. bullatarudis*) and how these preferences vary across three different fish stocks over time based on existing experimental data. The proposed MSM is developed to improve on previous estimates of survival probabilities given fish sex, fish size, fish stock and parasite strain. We further quantify and compare the virulence (measured by rate of host mortality and recovery) over time and estimate other relevant epidemiological parameters (mean time of host to remain infected and probability of infected host to either recover or die across the covariates).

## Methods

### Experimental data

The data used here are from the experimental study of Cable and van Oosterhout [45], subsequently used as the basis of an agent-based model in van Oosterhout [53]. Briefly, cultures of three different *Gyrodactylus* strains were used to infect three different fish stocks: Ornamental Stock (OS), Lower Aripo River fish (LA) and Upper Aripo River fish (UA); 157 guppies in total, in a full factorial design to give nine different host-parasite combinations, with 13–22 replicates per combination. Two out of the three parasite strains were *Gyrodactylus turnbulli*, a laboratory-bred strain (*Gt3*) and a wild *turnbulli* strain obtained from guppies caught in the Lower Aripo River, Trinidad (*Gt*), whereas the third strain was *G. bullatarudis*, also a wild type obtained from hosts in the Lower Aripo River. Both male (68) and female (89) individually isolated guppies were used for the experiment and maintained under constant environmental conditions ($25\pm 0.5 \,^\circ$C; 12h light/12h dark regime). All tanks and containers were kept in a randomised block design to reduce common environmental effects.

The fish considered in the experiment were naive and thus bred under parasite-free conditions. Each fish was then infected with two parasites at time 0, and parasites were counted every 48 h over a 17-day infection period. For each fish, the number of parasites was recorded across eight different body regions (tail fin, lower body, upper body, anal fin, dorsal fin, pelvic fins, pectoral fins and head). Survival data describing the various host infection statuses (remain infected, recovered from the infection or died) over time were extracted from the empirical data for the multi-state modelling. The number of surviving fish (with or without host infection loss) and dead fish across the nine different host-parasite groups over time from days 1 to 17 is tabulated in Appendix 1.

### Statistical analyses

#### Summary

**Fig. 1:**
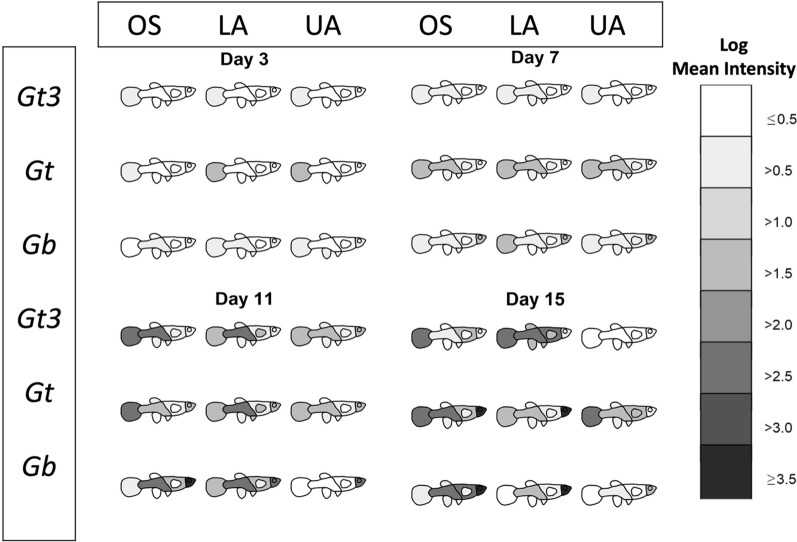
The movement of three different gyrodactylid parasites species/strains (*Gt3*, *Gt* and *Gb*) across eight host body parts (tail, lower body, anal fin, pelvic fins, dorsal fin, upper body, pectoral fins, head) of different fish stocks (OS, LA and UA stocks) at four time points. The degree of blackness indicates higher mean intensity (on log scale) over surviving fish

**Fig. 2:**
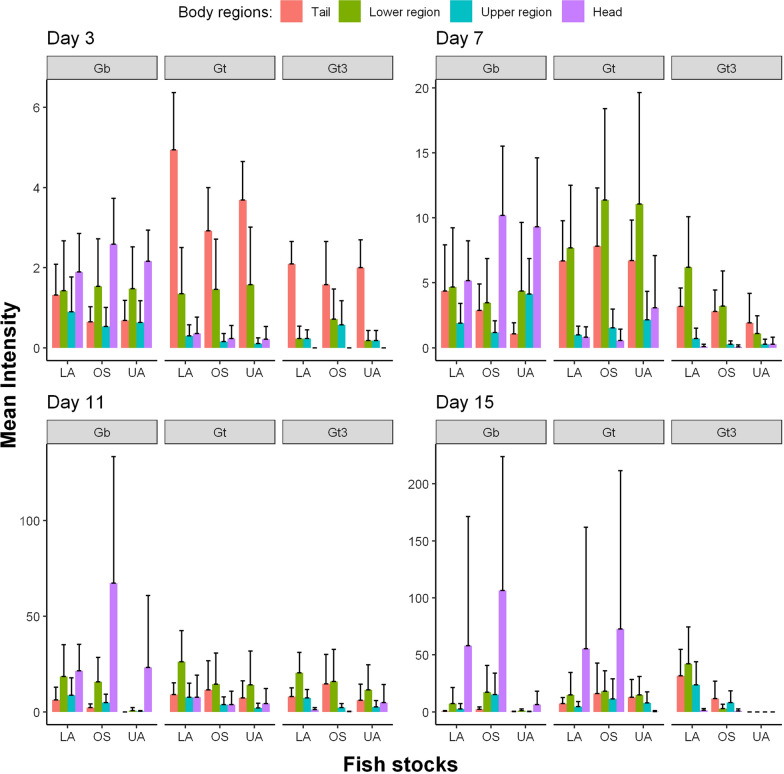
Mean intensities (with corresponding 95% confidence intervals) of three gyrodactylid strains (*Gt3*, *Gt* and *Gb*) at four main body regions (tail, lower region, upper region and head) across three fish stocks (OS, LA and UA stocks) over surviving fish and across time

All analyses were carried out using R version 3.6.3 [57]. Images of fish were produced in Gimp software version 2.10.12 [58] and outlined in R. Two graphical summaries of the data were produced. These are available in full in the Additional file 1: Fig. S1 and Additional file 2: Fig. S2, with examples given in Figs. 1 and 2. For the first summary (Fig. 1 and Additional file 1: Fig. S1), the shading represents the log mean intensity of parasites over surviving fish. The number of surviving fish for the nine different host-parasite groups (obtained from the fully crossed design of the three parasite strains and three different host populations) generally decreased (slowly) over time from days 1 to 17 (refer to Appendix 1). For the second graphical summary (Fig. 2 and Additional file 2: Fig. S2), the eight body regions of the fish were recategorized into four: tail, lower region (comprising of the lower body, anal fin, pelvic fins and dorsal fin), upper region (made up of the upper body and pectoral fins) and the head. This re-categorisation allowed us to visually and statistically assess any caudal-rostral preference of the three parasite strains on the three fish stocks more effectively over the study period because of low parasite numbers observed on the fish fins (anal fin, pelvic fins, dorsal fin and pectoral fin).

#### Multivariate Kruskal-Wallis test for parasite distribution comparison across host body regions

The multivariate Kruskal–Wallis test (MKW) is a multivariate extension of the distribution-free univariate Kruskal-Wallis test [59]. We used it to test the null hypothesis that distribution of parasite number at the four body regions (tail, lower region, upper region and head) is equal for the different host-parasite combinations at each observed time point.

Let $Y_{ij}$ be a vector of the number of parasites at the four body regions for the *j*th fish from the *i*th group (host-parasite combination), where $i=1,2,3,\cdots ,9$ and $j=1,2,3,\cdots ,n_{i}$. Let $R_{ij}$ be the rank corresponding to $Y_{ij}$ calculated element-wise (ties are assigned a mean rank) and ${\bar{R}}_{i}=\sum \nolimits _{j=1}^{n_i}{\frac{R_{ij}}{n_i}}$ then $E({\bar{R}}_{i})=m=\frac{n+1}{2}$ under $H_0$; where $n=\sum \nolimits _{i=1}^{9}{n_i}$ is the total number of fish ($n=157$), ${\bar{R}}_{i}$ is the mean rank for each *i*th group and ${n_i}$ is the number of fish in group *i*. The vector $U_{i}=(\bar{R}_{i1}-m,\bar{R}_{i2}-m,\bar{R}_{i3}-m,\bar{R}_{i4}-m)^\prime$ denotes the average ranks for the *i*th group corrected for *m* for each variate (body regions). The pooled within-group covariance matrix is estimated as

1 $$\begin{aligned} V=\frac{1}{n-1}\sum _{i=1}^{9}\sum _{j=1}^{n_i}(R_{ij}-m{1})(R_{ij}-m{1})^\prime \end{aligned}$$

where $R_{ij}=(R_{ij1},R_{ij2},R_{ij3},R_{ij4})^{\prime }$ and $1=(1,1,1,1)^{\prime }$. The MKW test statistic (${\mathcal {W}}$), given as

2 $$\begin{aligned} {\mathcal {W}}=\sum _{i=1}^{9}{n_i}{U_i}^{\top }{V^{-1}}{U_i} \sim {\chi }^{2}_{k(g-1)}, \end{aligned}$$

is approximately (asymptotically) chi-squared with $k(g-1)$ degrees of freedom, where $k=4$ and $g=9$ [59]. After performing the MKW, the univariate Kruskal-Wallis test (UKW) was used to further compare the distribution of parasites at each of the four body regions for each parasite strain (*Gt3*, *Gt* and *Gb*) across the fish stocks (OS, LA and UA) at each time point (days 1 to 17). A Bonferroni-Dunn’s post-hoc test was finally applied for pairwise comparisons of the parasite distribution between the different parasite-fish combinations over time. The caudal-rostral preference of the three parasite strains on the three fish stocks was statistically inferred from these tests (testing the niche partition hypothesis of *Gyrodactylus turnbulli* and *G. bullatarudis* for preferences at the caudal and head regions, respectively).

#### Multi-state Markov model for gyrodactylid infection progression

Individual fish after being infected can transition among three discrete host states—fish remains infected (state 1), fish alive with loss of infection (state 2) and fish dead (state 3)—over the observation period. Let $\{X_i(t); t \ge 0\}$ be the state of fish *i* over time. We suppose that $\{X_i(t)\}$ is a time-inhomogeneous Markov chain with transition rate matrix $Q(t)=\{q_{rs}(t)\}$ for $r,s=1,2,3$. For each $i=1,2, \cdots , 157$, we have observations $X_i=(X_{i0},X_{i1},\cdots ,X_{i9})$ at times $t_0=0$, $t_1=1$, $t_2=3$, $\cdots$ and $t_9=17$. The likelihood for the model parameters $\theta =\{q_{rs}(t)\}$ is given as

3 $$\begin{aligned} L(\theta )=\prod _{i=1}^{157}L_i(\theta |x_i) \end{aligned}$$

where $L_i(\theta |x_i)$ is the likelihood contribution for each fish *i* obtained as product of state transition probabilities

4 $$\begin{aligned} L_i(\theta |x_i)=\prod _{j=1}^{9}p_{x_{ij-1},x_{ij}}(t_{j-1},t_{j}) \end{aligned}$$

with $p_{x_{ij-1},x_{ij}}(t_{j-1},t_{j})=P\{X_i(t_{j})=x_{ij}|X_i(t_{j-1})=x_{ij-1} \}.$ We assumed that once a fish had lost its infection (state 2) or died (state 3), it could not be reinfected because of the experimental design (move back to state 1) and thus the corresponding rates are 0. Hence, the transition rate matrix *Q*(*t*) for the multi-state model with the three discrete host states is given aswhere $q_{12}(t)>0$ and $q_{13}(t)>0$ are the rates at which an infected fish loses its infection and dies at time *t* respectively. Here, we modelled the rate matrix *Q*(*t*) as a piecewise constant function with change points $t_1,t_2,\cdots ,t_{8}$. For $t{\in }[t_{j-1},t_j)$, we write $Q(t)=Q_j$. The transition probability matrix is

5 $$\begin{aligned} P(s,t)= \big (P_{ij}(s,t) \big )_{ij}=(P \big (X(t)=j|X(s)=i \big ) \big )_{ij}=e^{\int _s^{t} Q(u)du} \end{aligned}$$

The likelihood function for the model parameters is estimated using a maximum likelihood method, fitted using the *msm* package in R [60].

#### Estimating the probability of transition and virulence given covariates

We examine how variables such as fish sex, fish size, fish stock and parasite strain may affect the transition rates *Q*(*t*). Let $z_i=\{z_{i1},z_{i2},z_{i3}, z_{i4}\}$ be the realized values of the covariates (fish sex, fish size, fish stock and parasite strain) for fish *i*. Then, the transition rate matrix entries $q_{rs}(t)$ for $r, s= 1, 2, 3$ and $t{\in }[t_{j-1},t_j)$ were taken as

6 $$\begin{aligned} q_{rs}(t, z_i)={q_{rsj}^{(0)}}{exp(\beta _{rs1}z_{i1} + \beta _{rs2}z_{i2}+ \beta _{rs3}z_{i3}+ \beta _{rs4}z_{i4})} ={q_{rsj}^{(0)}}{exp(\beta _{rs}^{T}{z_i})} \end{aligned}$$

where ${q_{rsj}^{(0)}}$ is baseline intensity; $\beta _{rs}$ is a parameter vector. The likelihood is then maximized over ${q_{rsj}^{(0)}}$ and the regression coefficients $\beta _{rs}$, for $r=1$ and $s=2,3$. The hazard ratios (HR) corresponding to each covariate are $exp(\beta _{rs})$, for $r=1$ and $s=2,3$. The transition probabilities were estimated from $q_{rs}(t, z_i)$ using Eq. 5. Given the four predictors (fish sex, fish size, fish stock and parasite strain) and two possible transitions from state 1 to either state 2 ($q_{12}$) or state 3 ($q_{13}$) in the proposed multi-state Markov model (defined by Eq. 6), there are $16^2$ (or 256) possible variable permutations or models (which includes transitions independent of the underlying covariates).

A systematic variable and model selection was carried out using both Akaike information criterion (AIC) and Bayesian information criterion (BIC) statistics (due to the relative advantages of the two model selection criteria), where all possible variable permutations or models were considered. The AIC statistic assesses the model’s goodness of fit while reducing the complexity of the underlying parameters, whereas the BIC statistics penalise adding more parameters or strongly penalise free parameters compared to the AIC statistic. According to Kuha [61], effective model selection can be achieved by using both AIC and BIC statistics, predominantly to identify models favoured by both criteria, although the study’s methodological design, the main research questions and the belief of a true model and its applicability to the study are crucial factors in determining whether to utilise the AIC or BIC [62]. The best model (among identified parsimonious or highly predictive models) was finally chosen based on a likelihood ratio test (LRT) at a 5% significance level. Detailed results on the variable selection for the multi-state model and its R codes (for reproducibility of results) can be found via the GitHub URL link: github.com/twumasiclement/Spatial-Temporal-Parasite-Dynamics.

Let $T_1$ be the time spent in state 1, given that the fish or the process is in state 1 at time 0. Then, the mean sojourn time in state 1 is given as

7 $$\begin{aligned} E(T_1)= \sum _{j=1}^{\infty } E(T_1|\text {leave in period }j) \times \text {P(leave in period )j}, \end{aligned}$$

where

8 $$\begin{aligned} E(T_1\mid \text {leave in period }j)=t_{j-1}+E(S_j|S_j\le t_j-t_{j-1}) \end{aligned}$$

with

$$\begin{aligned} S_j \sim \exp (q_{12}(j,z_i)+q_{13}(j,z_i)), \end{aligned}$$

and $E(S_j|S_j\le t_j-t_{j-1})$ is given by Eq. 11 according to Theorem 1. In Eq. 7, the probability that the process leaves in period *j*, denoted by $\text {P(leave in period j)}$, is computed such that

$$\begin{aligned} \text {P(leave in period j)}= \left\{ \begin{array}{ll} P(S_j \le t_j-t_{j-1}),&{} j=1\\ \left[ 1- \sum _{j^{\prime }=1}^{j-1} \text {P(leave in period} j^{\prime }) \right] \times P(S_j \le t_j-t_{j-1}), &{} 2 \le j \le 7\\ 1- \left[ \sum _{j^{\prime }=1}^{7} \text {P(leave in period} j^{\prime }) \right] , &{} j \ge 8 \\ \end{array}\right. \end{aligned}$$

with

$$\begin{aligned} P(S_j\le {t_j-t_{j-1}} )=1-\mathrm{e}^{-({{q_{12}(j,z_i)+q_{13}(j,z_i)})(t_j-t_{j-1})} } \quad \quad \text {for} \quad j \ge 1 \end{aligned}$$

in accordance to Eq. 10 under Theorem 1.

##### Theorem 1

Let $S_j$ be the time spent by infected fish during period *j*. Suppose that $S_j \sim \exp \left( q_{12}(j,z_i)+q_{13}(j,z_i) \right)$ with probability density

$$\begin{aligned} f(S_j)= \left[ q_{12}(j,z_i)+q_{13}(j,z_i)\right] \mathrm{e}^{-(q_{12}(j,z_i)+q_{13}(j,z_i))S_j}, \quad S_j>0 \end{aligned}$$

where $q_{12}(j,z_i)$ and $q_{13}(j,z_i)$ are the transition rates from state 1 to state 2 and 3, respectively, given the covariates $z_i$ for fish *i*, such that

$$\begin{aligned} E(S_j)=\frac{1}{{q_{12}(j,z_i)+q_{13}(j,z_i)}}. \end{aligned}$$

Then,

9 $$\begin{aligned} E \left[ S_j \mathbbm {1}_{\{S_j \le {t_j-t_{j-1}}\}} \right] =E(S_j) - \left[ t_j-t_{j-1}+E(S_j) \right] \mathrm{e}^{-({{q_{12}(j,z_i)+q_{13}(j,z_i)})(t_j-t_{j-1})} } \end{aligned}$$

and

10 $$\begin{aligned} P(S_j\le {t_j-t_{j-1}} )=1-\mathrm{e}^{-({{q_{12}(j,z_i)+q_{13}(j,z_i)})(t_j-t_{j-1})} }. \end{aligned}$$

For the mathematical proof to Theorem 1, see Appendix 2. From Theorem 1, it can be deduced that

11 $$\begin{aligned} \begin{aligned} E(S_j|S_j\le t_j-t_{j-1})&=\frac{E \left[ S_j \mathbbm {1}_{\{S_j \le {t_j-t_{j-1}}\}} \right] }{P(S_j\le {t_j-t_{j-1}} )} \\&= \frac{E(S_j) - \left[ t_j-t_{j-1}+E(S_j) \right] \mathrm{e}^{-({{q_{12}(j,z_i)+q_{13}(j,z_i)})(t_j-t_{j-1})} } }{1-\mathrm{e}^{-({{q_{12}(j,z_i)+q_{13}(j,z_i)})(t_j-t_{j-1})} }}, \end{aligned} \end{aligned}$$

where

$$\begin{aligned} E(S_j)=\frac{1}{{q_{12}(j,z_i)+q_{13}(j,z_i)}}. \end{aligned}$$

Also, given the fish or process is in state 1, then the probability of moving to state 2 or 3 next is given as

12 $$\begin{aligned}&P(\text {transition from state 1 to s}| \text {leave state 1}) \nonumber \\&\quad =\sum _{j=1}^{\infty }P(\text {transition from state 1 to s}| \text {leave in period }j) \times P(\text {leave in period} j), \end{aligned}$$

where

$$\begin{aligned} P(\text {transition from state 1 to s}| \text {leave in period }j)=\frac{q_{1s}(j,z_i)}{q_{12}(j,z_i)+q_{13}(j,z_i)} \end{aligned}$$

for $s=2,3$. We assume that $q_{12}(t,z_i)=q_{12}(15,z_i)$ and $q_{13}(t,z_i)=q_{13}(15,z_i)$ for $t \ge 15$.

## Results

### Parasite microhabitat preferences

Fish heatmaps (Fig. 1 and Additional file 1: Fig. S1) depict variations in parasite distribution across eight body regions (caudal fin, lower body, upper body, anal fin, pelvic fin, dorsal fin, pectoral fin and head) over time for each gyrodactylid strain (*Gt3*, *Gt* and *Gb*) on the different fish stocks (OS, LA and UA). *Gt3* showed a clear preference for the caudal fin and lower body, with higher mean intensities on OS and LA fish than on the UA stock from day 7 until the end of the infection period. By day 15, all the UA fish had lost the *Gt3* infection. Similarly, *Gt* was more abundant on the tail and lower body until day 13, but switched to a head preference among only OS and LA populations on day 15. In contrast, *Gb* showed a clear rostral preference from day 7 onwards, a preference strongest in OS$\ge$LA$\ge$UA fish stocks until the end of the infection period.

When comparing just four body regions of the fish (tail, lower region, upper region and head), the peak time of infection varied spatially across parasite strains and fish stocks (Table 1; Fig. 2). On day 15, higher mean intensities were recorded on the head for both *Gt* and *Gb* on OS fish stock. Also for *Gb* on the same fish stock, a higher number of parasites occurred on the head between days 9 and 17 compared to any other body region or host-parasite combinations (Fig. 2 and Additional file 2: Fig. S2). Parasite distributions varied at the four body regions across the nine host-parasite combinations (Fig. 2) from days 1 to 15 (MKW, $71.25{\le }W{\le }168.57$, $df=32$, $p<0.001$), but not on day 17 ($W=38.12$, $df=32$, $p=0.211$). Only the parasite distribution at the tail and head respectively differed significantly across the nine host-parasite combinations from days 1 to 5 and on day 9 ($p\le 0.001$). However, parasite distribution differed significantly among groups on the lower body region on days 7 and 11 (UKW, ${17.12\le }H{\le }17.74$, $df=8$, $0.023{\le }p{\le }0.029$), tail on days 7 and 15 (${19.49\le }H{\le }24.93$, $df=8$, $0.002{\le }p{\le }0.012$) and head on days 7, 11 and 13 ($21.22{\le }H{\le }47.36$, $df=8$, $0.001<p{\le }0.007$).

**Table 1:** Peak time of gyrodactylid infection (in days) across three different parasite strains (*Gt3*, *Gt* and *Gb*) and three fish stocks (OS, LA and UA) for four body regions

Parasite strains	Fish	Tail	Lower region	Upper region	Head
*Gt3*	OS	11	11	15	15
	LA	15	15	15	9
	UA	11	11	13	11
*Gt*	OS	17	13	15	15
	LA	11	11	11	15
	UA	9	9	17	9
*Gb*	OS	7	13	17	15
	LA	11	11	11	15
	UA	5	7	9	13

From the Bonferroni-Dunn tests, there were significant pairwise differences in parasite distribution at the tail between all *Gb* groups (*Gb*-OS, *Gb*-LA and *Gb*-UA) and *G. turnbulli* strains on the fish stocks (with the exception of *Gt3* on OS) during day 1 of infection ($0.001< p\le$0.016). However, there was no significant difference in parasite distribution of the *G. turnbulli* strains at the tail across the three fish stocks over time; with the exception of days 3 and 15, between *Gt3*-OS and *Gt*-LA groups ($p=0.019$) as well as between *Gt3*-LA and *Gt3*-UA groups ($p=0.037$). On days 3 and 5, parasite distribution at the tail was significantly different ($0.001<p\le$0.036) between all *Gb* groups and *Gt* groups with the exception *Gt*-OS for day 3 and *Gt*-UA for day 5. Parasite distribution at the tail on day 7 was significantly different between *Gb*-UA and *Gt* groups (*Gt*-OS and *Gt*-UA), whilst a significant difference was found between *Gb-UA* and *Gt* groups (*Gt*-LA and *Gt*-UA). Nevertheless, there was no significant difference between groups of the *G. turnbulli* strains and *G. bullatarudis* from day 15 till the end of the infection period. Significant difference in parasite distribution on the lower region only occurred on day 7 between *Gt*-OS and *Gb*-UA groups ($p=0.039$) and on day 11 between *Gb*-UA and *Gt*-LA groups ($p=0.014$). Nonetheless, parasite distribution on the head was significantly different ($0.001<p\le 0.013$) between each of the *G. bullatarudis* groups (*Gb*-OS and *Gb*-UA) and all the *G. turnbulli* groups on day 1. But from days 3 to 5, significant pairwise difference ($0.001<p\le 0.046$) was found between all *Gb* groups and *turnbulli* strains for all fish stocks respectively at the head. However, apart from *Gb*-OS group that still showed significant difference with all *G. turnbulli* groups on day 7 ($0.001<p\le 0.016$), *Gb*-LA and *Gb*-UA rather showed significance difference ($0.001<p\le 0.037$) with *Gt3*-OS and *Gt3*-LA. Nevertheless, *Gb* on OS showed difference significantly ($0.001<p\le 0.013$) on the head with *Gt3* on OS and LA stocks as well as *Gt* on LA population during day 9, whereas two groups of *Gb* (on OS and LA stocks) had significant difference with only *Gt3* on OS fish population during day 11 of the infection period. On day 13, there was significant difference in parasite distribution on the head between *Gb* and *Gt* on ornamental fish only.

### Multi-state Markov model of gyrodactylid infection progression

**Fig. 3:**
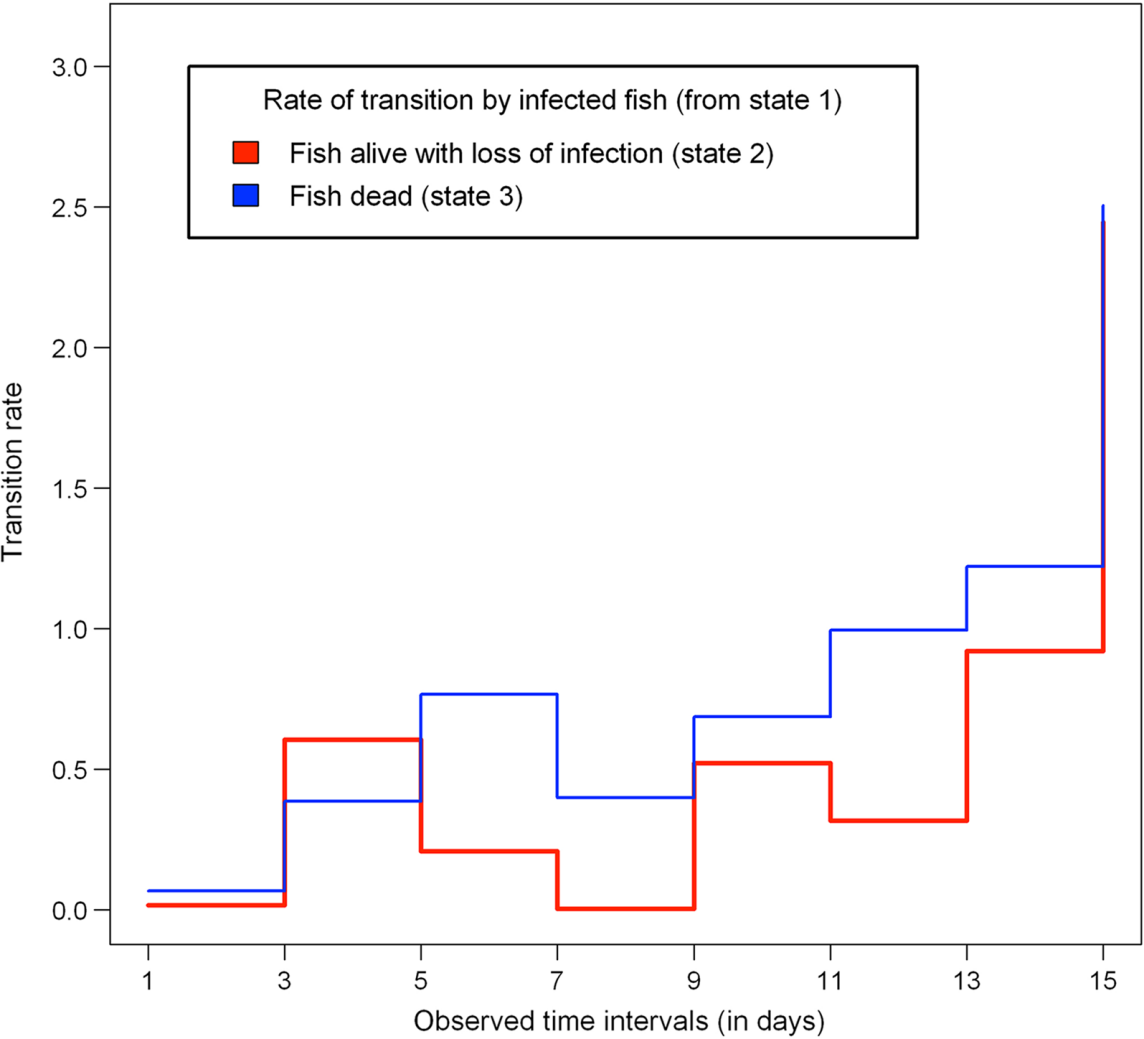
Piecewise-constant plot of estimated baseline transition rates from infected host state to uninfected and dead states at different observed time intervals in the time-inhomogeneous multi-state Markov model

**Fig. 4:**
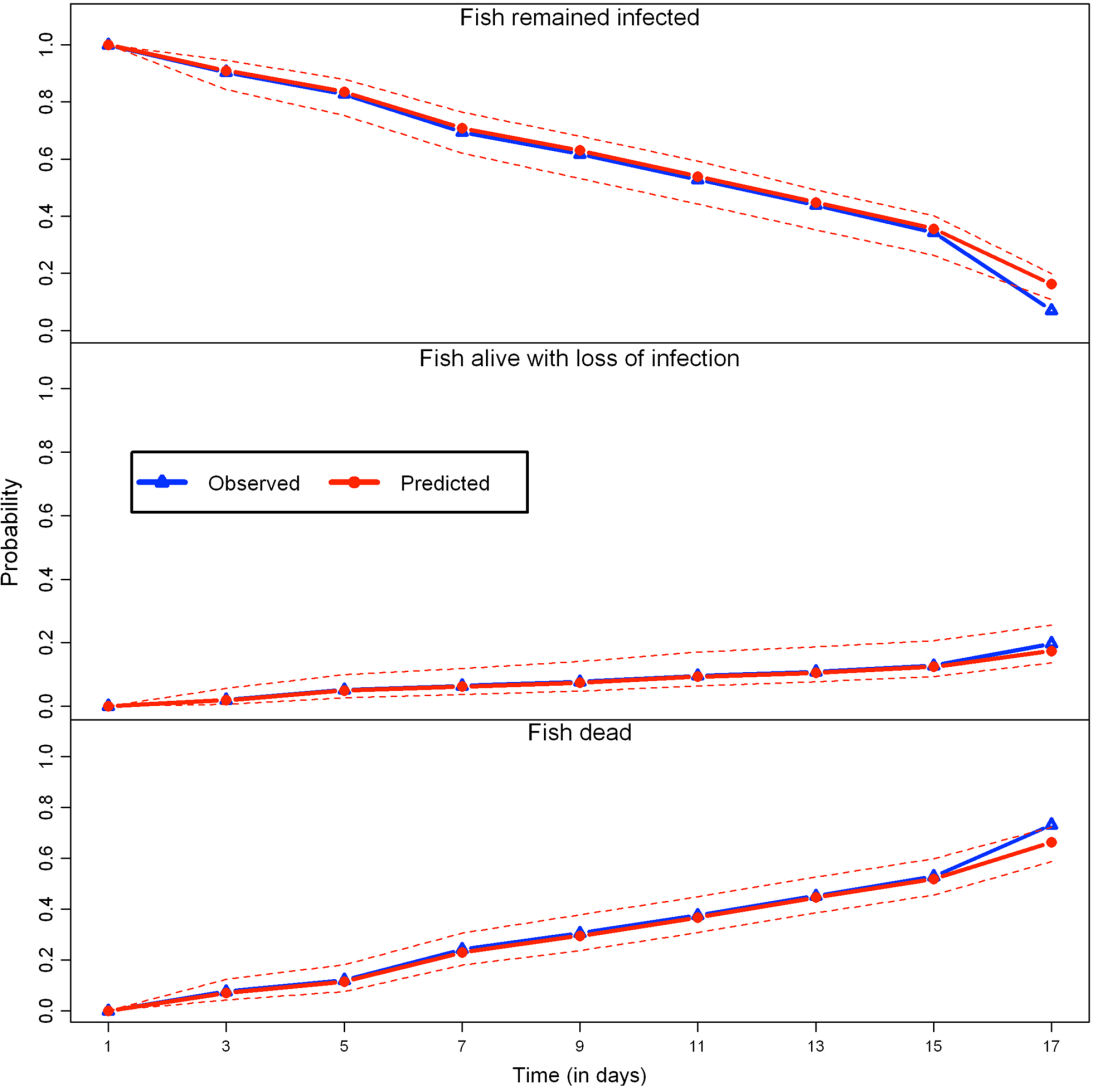
Comparison between observed and expected proportions of fish that will remain in each host infection state from days 1 to 17 after the onset of gyrodactylid infection based on the fitted multi-state Markov model (mean absolute percentage error = 7.85%)

We used the time-inhomogeneous multi-state Markov model to examine the significant determinants of fish survival (fish sex, fish size, fish stock and parasite strain). The estimated hazard ratios (HR) corresponding to each significant predictor of the fitted model are summarized by Table 2. Figure 3 shows how the baseline transition rates from the infected state (state 1) to uninfected (state 2) and dead (state 3) states changed over the observed time intervals. Figure 4 shows that the fitted multi-state model gives a very good fit to the proportion of fish that will remain in each host infection status from the onset of infection to the end of the study period.

**Table 2:** Estimated hazard ratios (HR) from the multi-state Markov model across significant predictors (fish sex, fish size, fish stock and parasite strain) with their respective 95% confidence intervals (CI)

Covariates	Transitions	HR	Lower CI	Upper CI	*P*-value
Fish size	$1 \rightarrow 2$	0.87	0.76	0.99	0.037$^*$
Fish sex					
Male (Ref: Female)	$1 \rightarrow 3$	1.52	1.04	2.22	0.031$^*$
Fish stock					
OS (Ref: UA)	$1 \rightarrow 3$	0.24	0.14	0.39	$<0.001^*$
LA (Ref: UA)	$1 \rightarrow 3$	0.39	0.25	0.61	$<0.001^*$
Parasite strain					
*Gt3* (Ref: *Gt*)	$1 \rightarrow 3$	1.65	1.03	2.65	0.037$^*$
*Gb* (Ref: *Gt*)	$1 \rightarrow 3$	1.64	1.02	2.62	0.039$^*$

$$^*$$ Statistically significant

**Fig. 5:**
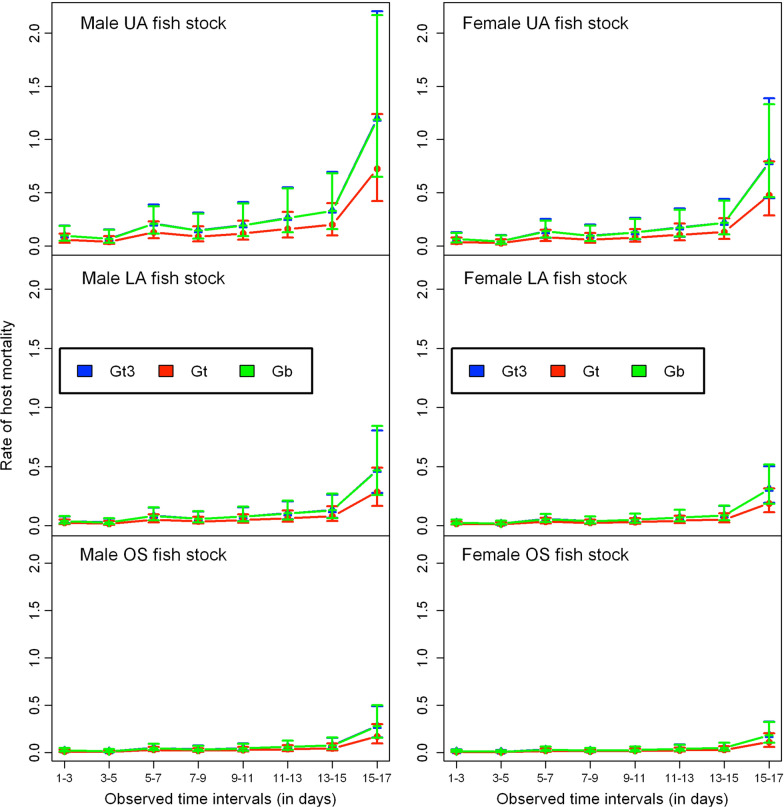
Predicted host mortality rates of parasite strains (*Gt3*, *Gt* and *Gb*) on the fish stocks (OS, LA and UA stocks) over time for both male and female fish respectively

**Fig. 6:**
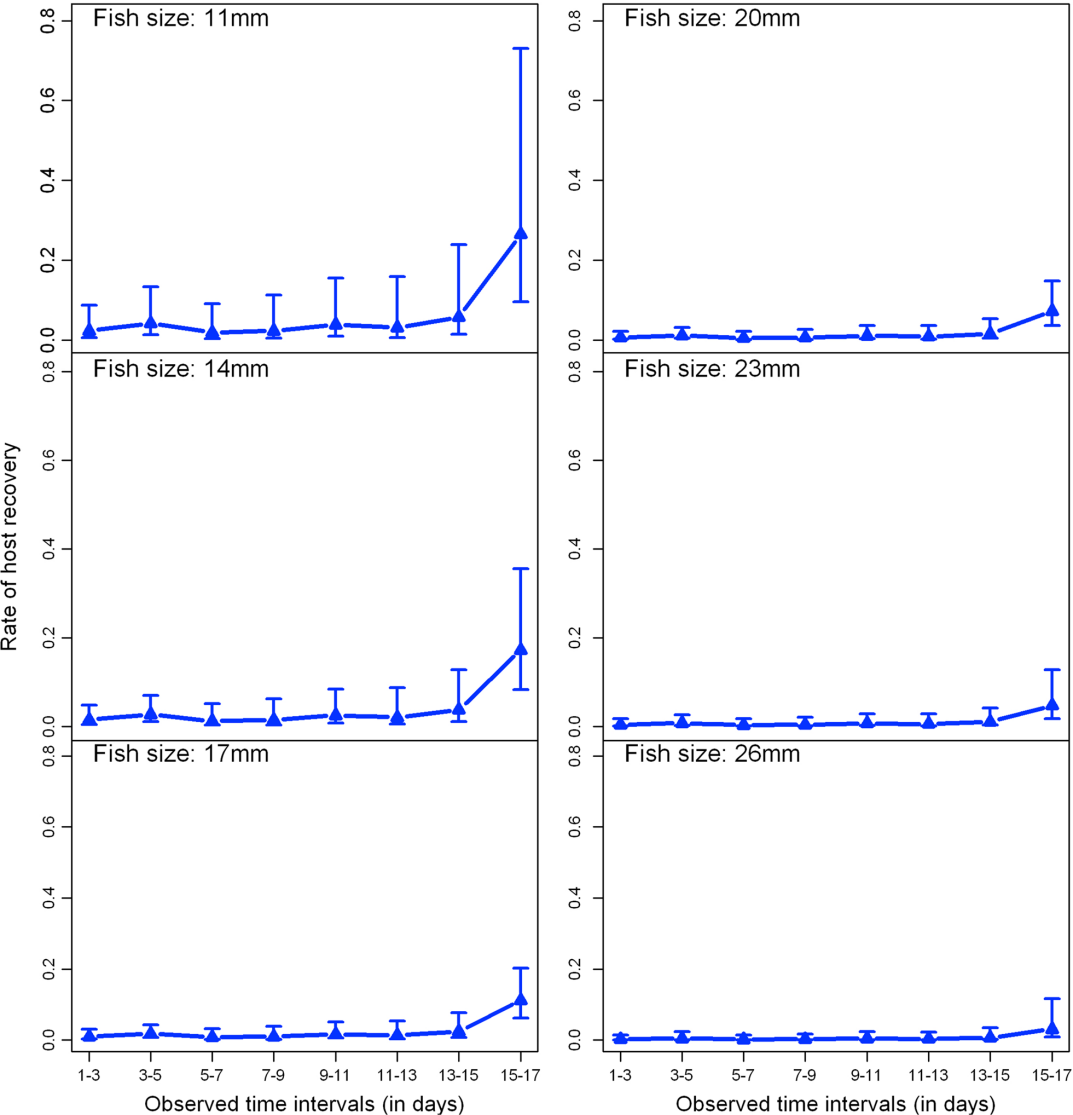
Predicted host recovery rates over time at different fish sizes (11, 14, 17, 20, 23 and 26 mm)

The likelihood of infected fish fighting off their infection was significantly influenced by fish size ($\hbox {HR}=0.87$, 95% $\hbox {C.I}=0.76$-0.99, $p=0.037$), such that larger fish are less likely to clear off their infection. Fish sex, fish stock and parasite strain did influence the likelihood of infected fish dying, but not parasite extinction. Infected male fish were 52% more likely to die compared to female fish ($\hbox {HR}=1.52$, 95% $\hbox {C.I}=1.04$-2.22, $p=0.031$). The risk of death from the gyrodactylid infection among the OS fish ($\hbox {HR}=0.24$, 95% $\hbox {C.I}=0.14$-0.39, $p<0.001$) was 76% less likely compared to UA fish stock. LA fish ($\hbox {HR}=0.39$, 95% $\hbox {C.I}=0.25$-0.61, $p<0.001$) were 61% less likely to die from gyrodactylid infections relative to UA fish. Based on estimated hazard ratios, the rate of fish survival from the gyrodactylid infections was higher among OS stock, followed by LA stock and then UA stock. Fish infected by laboratory strain of *G. turnbulli* ($\hbox {HR}=1.65$, 95% $\hbox {C.I}=1.03$-2.65, $p=0.037$) were 65% more likely to die compared to the wild strain. The wild *G. bullatarudis* strain ($\hbox {HR}=1.64$, 95% $\hbox {C.I}=1.02$-2.62, $p=0.039$) was also 64% more likely to kill fish compared to the wild *G. turnbulli* strain. The estimates of the hazard ratios corresponding to *Gt3* and *Gb* relative to wild *G. turnbulli* strain suggest that there is no significant difference in the likelihood of fish mortality between *Gt3* and *Gb* strains. We quantified parasite virulence by estimating the rates of both host mortality (Fig. 5) and host recovery (Fig. 6) over time using the fitted multi-state Markov model.

We estimated the mean sojourn time in state 1 (the average amount of time fish can remain infected) and the probability of next transition from the infected state (state 1) to either recovery (state 2) or dead state (state 3) across all significant predictors (fish sex, fish size, fish stock and parasite strain) of the fitted multi-state Markov model. For any strain of gyrodactylid, large ornamental female fish remained infected longer than fish with any other attributes (Table 3). Fish infected with the wild *G. turnbulli* strain on average remained infected longer than fish infected with *Gt3* or wild *G. bullatarudis* strains before either recovering or dying, irrespective of the fish size, stock and sex. The mean time for fish to remain infected with any parasite strain before fighting off their infection or dying was between 6 and 14 days. An infected fish had a higher probability of dying than recovering from the infection irrespective of the type of gyrodactylid infection, fish stock, sex and size (Table 4). Large male fish were more likely to die than small or medium-sized male or female fish of any size, whereas the chance of host recovery was higher among OS fish stock compared to the Trinidadian fish stocks. The fish infected with wild *G. turnbulli* strain had a greater probability of fighting off their infections than fish infected with either *Gt3* or *Gb* strain.

**Table 3:** Mean sojourn time (in days) for fish to remain infected across significant predictors (fish sex, fish size, fish stock and parasite strain) based on the fitted multi-state Markov model

Parasite strain	Fish stock	Male fish			Female fish		
		Small	Medium	Large	Small	Medium	Large
		(11 mm)	(17 mm)	(26 mm)	(11 mm)	(17 mm)	(26 mm)
*Gt3*	OS	10.69	11.33	11.79	11.40	12.13	12.64
	LA	9.52	10.03	10.40	10.49	11.12	11.56
	UA	6.78	7.04	7.22	8.06	8.43	8.69
*Gt*	OS	11.52	12.26	12.79	12.01	12.81	13.37
	LA	10.67	11.31	11.76	11.38	12.11	12.62
	UA	8.32	8.71	8.99	9.49	10.00	10.36
*Gb*	OS	10.71	11.35	11.81	11.42	12.14	12.66
	LA	9.54	10.06	10.43	10.51	11.14	11.58
	UA	6.81	7.07	7.25	8.09	8.46	8.72

**Table 4:** Probability of next transition from the infected state 1 to either the recovery state 2 or the dead state 3 across significant predictors (fish sex, fish size, fish stock and parasite strain) based on the fitted multi-state Markov model

Parasite strain	Fish stock	Male fish						Female fish					
		Small		Medium		Large		Small		Medium		Large	
		(11 mm)		(17 mm)		(26 mm)		(11 mm)		(17 mm)		(26 mm)	
		*p*_12_	*p*_13_	*p*_12_	*p*_13_	*p*_12_	*p*_13_	*p*_12_	*p*_13_	*p*_12_	*p*_13_	*p*_12_	*p*_13_
*Gt3*	OS	0.460	0.540	0.357	0.643	0.266	0.734	0.565	0.435	0.460	0.540	0.358	0.642
	LA	0.338	0.662	0.249	0.751	0.177	0.823	0.436	0.564	0.335	0.665	0.247	0.753
	UA	0.177	0.823	0.122	0.878	0.082	0.918	0.238	0.762	0.168	0.832	0.116	0.884
*Gt*	OS	0.586	0.414	0.481	0.529	0.378	0.622	0.683	0.317	0.587	0.413	0.483	0.517
	LA	0.457	0.543	0.355	0.645	0.264	0.736	0.562	0.438	0.456	0.543	0.355	0.645
	UA	0.253	0.747	0.179	0.821	0.124	0.875	0.335	0.665	0.246	0.754	0.176	0.824
*Gb*	OS	0.463	0.537	0.359	0.641	0.268	0.732	0.567	0.433	0.462	0.538	0.360	0.640
	LA	0.340	0.660	0.250	0.750	0.179	0.821	0.438	0.562	0.337	0.663	0.249	0.751
	UA	0.178	0.822	0.123	0.877	0.083	0.917	0.240	0.760	0.169	0.831	0.116	0.884

## Discussion

### Insights into the gyrodactylid-guppy system

In this study, we built on previous studies of the infrapopulation dynamics of three different gyrodactylid strains (two strains of *G. turnbulli* and one strain of *G. bullatarudis*) among three different fish stocks (OS, LA and UA stocks) in relation to parasite habitat preference, host survival and parasite virulence (see [45, 48, 49, 56]). We have confirmed for the first time that the microhabitat preferences of the *G. turnbulli* (laboratory and wild type) and *G. bullatarudis* strains depend on the type of host and can change over time for the wild *G. turnbulli* strain (with the help of a multivariate ranked-based distribution-free test and associated post-hoc tests). With an extension to the traditional survival models, we have been able to include host recovery as another absorbing state, and fish sex was identified as a significant factor of host survival compared to previous study of this biological system [45]. We also estimated for the first time, the average duration that fish can remain infectious and the probability that infected fish will either recover or die from each of the three parasite strains across the three guppy populations, sexes and different fish sizes (small, medium and large sizes).

The captive-inbred *G. turnbulli* strain preferred the tail of three different fish stocks (Ornamental, Lower Aripo River and Upper Aripo River stocks), whereas the wild *G. turnbulli* initially preferred the tail but then switched to the head. The wild *Gyrodactylus bullatarudis* consistently showed a rostral preference on all fish. The mean intensity of parasites was higher on OS and LA fish than UA stocks across all body regions over time, probably related to the higher mortality of the UA fish. Lower numbers of parasites on the pectoral, pelvic, dorsal and anal fins compared to the tail, lower body, upper body and head regions might be affected by fish being maintained in isolation or due to difference in the surface area of these body regions. Individual host isolation meant there was no opportunity for host-to-host transmission to occur via the fins (as suggested by [48]). Thus, the parasites might be making a behavioural decision to enhance their fitness in response to the absence of alternative hosts and or reduce competition at small-sized body regions over time. The peak time to infection varied spatially across parasite strains and fish stocks. Such variation likely represents a trade-off between successful parasite exploitation and the host’s localised immune response (reviewed by [34]). Parasite distribution on infected hosts could also be driven by multiple abiotic and biotic factors [50–52, 54].

The fitted multi-state model revealed that fish sex, fish stock and parasite strain influenced fish mortality. LA and OS fish stocks survived for longer than UA fish. For this gyrodactylid-fish system, the current study revealed that a longer period of host infection leads to a higher chance of host recovery and a smaller chance of host mortality. The OS guppy population was infectious longer than the Trinidadian fish stocks (LA and UA fish) based on the estimated average duration of infection. However, the OS guppies had a higher chance of host recovery compared to the LA and UA fish stocks, potentially due to superior innate immune defences or immunocompetence towards single-species infections (as revealed by [63, 64]). The LA fish consistently had better parasite resistance than the UA stock across fish sex, parasite strain and different host sizes. Larger fish were infectious over a longer period than small or medium-sized fish, whereas female fish from all three guppy populations experienced a longer duration of infection than male fish. Fish infected by the wild strain of *G. turnbulli* on average remained infected longer with a higher probability of host recovery among all fish stocks and sexes.

As in the previous study, the laboratory strain of *G. turnbulli* and wild strain of *G. bullatarudis* were more likely to cause fish mortality than the wild strain of *G. turnbulli*, but we found that infected male fish were twice as likely to die relative to female fish. The main reason for this new finding of fish sex as a significant determinant of host mortality is the use of a multi-state model that is able to incorporate host mortality and recovery simultaneously. Other parasite-fish studies have identified fish sex as a significant factor of host mortality [65]. Only fish size significantly influenced the rate of infection loss, namely larger fish acquired more parasites as infections progressed resulting in low parasite extinction compared to smaller fish [53]. Nevertheless, it was found that the chance of host mortality was more likely to occur than host recovery irrespective of host size.

Parasite virulence, described in terms of host mortality and recovery, was significantly time dependent and generally increased towards the end of the infection period. Previously, *Gt3* was identified as causing most host deaths, followed by *G. bullatarudis* and then the wild *G. turnbulli*, but their respective host mortality rates were not quantified, nor did we previously consider how this changed over time, nor the effect of the different fish stocks [45]. Here, we found no significant difference in host mortality rates between *Gt3* and *Gb* parasite strains over time. Male fish from the three different guppy populations (OS, LA and UA stocks) consistently had a higher rate of host mortality than female fish stocks over time. This could be explained by the fact that the female fish are infectious longer than the male fish as revealed by the estimated mean sojourn time of infection; thus, the female host populations likely develop innate or adaptive host immunity faster than the male fish stocks over time.

### Wider mathematical implications of this study

The current study could inform the modelling and survival analyses of other biological systems where the entire infection history of an individual (or host) is of interest. Multi-state Markov models provide a robust approach to modelling almost any kind of longitudinal time-to-event data [20]. For multi-state processes that are misclassified or can only be viewed through a noisy marker, hidden Markov models can be implemented [25]. There is more extensive literature on different classes of multi-state Markov models and Markov extension models with specific applications to the modelling of fertility, infectious diseases, competing risks, disability, recurrent events, twin survival and alternating events (reviewed by [15]). However, they have been underused in most parasitological fields.

In the current multi-state Markov model, we could not include spatial information and other relevant information about parasite fecundity, age group (young or old parasite), parasite mortality, parasite mobility and host immune response. A more sophisticated (individual-based) stochastic simulation model would be needed to include these data to further understand the gyrodactylid-fish system. Future studies will examine host-to-host transmission to holistically understand the spread of gyrodactylid parasites and the host-parasite interactions among different populations of fish.

## Conclusions

In summary, we identified host-parasite strain-specific microhabitat preferences, discovered determinants of host survival and quantified host-specific parasite virulence based on both host mortality and recovery. The multi-state model was designed so that fish could not be reinfected after infection to match the experimental design, but this could be modified in future studies to include transmission. The multi-state Markov model and the rank-based multivariate Kruskal-Wallis test can be extended and adapted for studying other host-parasite interactions.

### Supplementary Information

**Additional file 1: Fig. S1.** Detailed visualisation of fish heatmaps over eight body regions of fish across parasite strains and fish stocks over time (from day 1 to 17).

**Additional file 2: Fig. S2.** Grouped barcharts showing variations in mean intensities at four main body regions of fish across parasite strains and fish stocks over surviving fish and across time (from day 1 to 17).

## Data Availability

The datasets generated and/or analysed during the current study are available from the corresponding author on reasonable request. The R codes used for all statistical analyses have been made publicly available for reproducibility of results via the URL link: https://github.com/twumasiclement/Spatial-Temporal-Parasite-Dynamics.

## Appendices

### Appendix 1: Number of surviving fish and dead fish for the nine different host-parasite groups over time from days 1 to 17

**Table:**

Days	*Gt3*			*Gt*			*Gb*			Total (*n*)
	OS	LA	UA	OS	LA	UA	OS	LA	UA	
*Fish alive with infection*										
Day 1	14	22	17	13	17	19	17	19	19	157
Day 3	13	20	13	11	16	16	16	19	16	140
Day 5	13	19	8	11	16	13	15	18	15	128
Day 7	13	18	4	11	14	11	14	14	10	109
Day 9	12	17	3	11	13	10	13	12	6	97
Day 11	12	15	3	10	13	6	11	10	3	83
Day 13	11	12	2	10	11	5	10	6	3	70
Day 15	9	10	0	7	10	5	7	4	2	54
Day 17	0	0	0	3	3	2	1	2	0	11
*Fish alive with loss of infection*										
Day 1	0	0	0	0	0	0	0	0	0	0
Day 3	1	2	0	0	0	0	1	0	1	5
Day 5	1	2	3	0	0	1	1	1	1	10
Day 7	1	1	3	0	0	1	1	1	2	10
Day 9	2	1	3	0	0	1	2	1	2	12
Day 11	2	1	3	1	0	1	4	1	2	15
Day 13	2	2	3	1	0	1	4	1	2	16
Day 15	3	2	3	2	0	1	5	2	2	20
Day 17	5	4	3	3	3	1	7	3	2	31
*Fish dead*										
Day 1	0	0	0	0	0	0	0	0	0	0
Day 3	0	0	4	2	1	3	0	0	2	12
Day 5	0	1	6	2	1	5	1	0	3	19
Day 7	0	3	10	2	3	7	2	4	7	38
Day 9	0	4	11	2	4	8	2	6	11	48
Day 11	0	6	11	2	4	12	2	8	14	59
Day 13	1	8	12	2	6	13	3	12	14	71
Day 15	2	10	14	4	7	13	5	13	15	83
Day 17	9	18	14	7	11	16	9	14	17	115

### Appendix 2: Mathematical proof of Theorem 1

#### Proof

For simplicity, let $S_j \sim \exp \left( \lambda \right)$ with probability density $f(S_j)= \lambda \mathrm{e}^{-\lambda S_j}, \quad S_j>0$ and $E(S_j)=\frac{1}{\lambda }$, where $\lambda =q_{12}(j,z_i)+q_{13}(j,z_i)$. Suppose $\alpha =t_j-t_{j-1}$, then

$$\begin{aligned} \begin{aligned} E \left[ S_j \mathbbm {1}_{\{S_j \le {\alpha }\}} \right]&=\int _{0}^{\alpha } S_jf(S_j)dS_j=\lambda \int _{0}^{\alpha }S_j\mathrm{e}^{-\lambda S_j}dS_j \\&=\lambda I, \quad \text {where} \quad I=\int _{0}^{\alpha }S_j\mathrm{e}^{-\lambda S_j}dS_j. \end{aligned} \end{aligned}$$

Considering the integral *I* and using integration by parts,

$$\begin{aligned} I=\int _{0}^{\alpha }S_j\mathrm{e}^{-\lambda S_j}dS_j=uv\biggr |_{0}^{\alpha }- \int _{0}^{\alpha }u^{\prime }v dS_j, \end{aligned}$$

where $$u=S_j$$, $$u^{\prime }=1$$, $$v^{\prime }=\mathrm{e}^{-\lambda S_j}$$ and $$v=-\frac{1}{\lambda }\mathrm{e}^{-\lambda S_j}$$.

$$\begin{aligned} \begin{aligned} \implies I&= -\frac{S_j}{\lambda }\mathrm{e}^{-\lambda S_j}\biggr |_{0}^{\alpha }+ \int _{0}^{\alpha }\frac{1}{\lambda }\mathrm{e}^{-\lambda S_j} dS_j= -\frac{\alpha }{\lambda }\mathrm{e}^{-\lambda \alpha } - \frac{\alpha }{\lambda ^2}\mathrm{e}^{-\lambda \alpha } +\frac{1}{\lambda ^2}\\&= -\frac{1}{\lambda }\mathrm{e}^{-\lambda \alpha } \left[ a+ \frac{1}{\lambda } \right] + \frac{1}{\lambda ^2}. \end{aligned} \end{aligned}$$

Hence,

$$\begin{aligned} \begin{aligned} E \left[ S_j \mathbbm {1}_{\{S_j \le {\alpha }\}} \right]&=\lambda I=\lambda \left( -\frac{1}{\lambda }\mathrm{e}^{-\lambda \alpha } \left[ \alpha + \frac{1}{\lambda } \right] + \frac{1}{\lambda ^2} \right) \\&=\frac{1}{\lambda }-\left[ \alpha + \frac{1}{\lambda } \right] \mathrm{e}^{-\lambda \alpha }= E(S_j)-\left[ \alpha + E(S_j) \right] \mathrm{e}^{-\lambda \alpha }. \end{aligned} \end{aligned}$$

Substituting for $\lambda$ and $\alpha$ gives the required Eq. 9 such that

$$\begin{aligned} E \left[ S_j \mathbbm {1}_{\{S_j \le {t_j-t_{j-1}}\}} \right] =E(S_j) - \left[ t_j-t_{j-1}+E(S_j) \right] \mathrm{e}^{-({{q_{12}(j,z_i)+q_{13}(j,z_i)})(t_j-t_{j-1})} }. \end{aligned}$$

Also, the required Eq. 10 can be obtained such that

$$\begin{aligned} \begin{aligned} P(S_j\le {t_j-t_{j-1}} )&=P(S_j\le \alpha )=\int _{0}^{\alpha }\lambda \mathrm{e}^{-\lambda S_j}dS_j \\&= \lambda \int _{0}^{\alpha }\mathrm{e}^{-\lambda S_j}dS_j=\lambda \left[ -\frac{1}{\lambda }\mathrm{e}^{-\lambda S_j}\right] _{0}^{\alpha }\\&=1- \mathrm{e}^{-\lambda \alpha }=1-\mathrm{e}^{-({{q_{12}(j,z_i)+q_{13}(j,z_i)})(t_j-t_{j-1})} }. \end{aligned} \end{aligned}$$

$$\square$$

## Abbreviations

AIC: Akaike information criterion
BIC: Bayesian information criterion
*Gb*: Wild *Gyrodactylus bullatarudis* strain
*Gt*: Wild *Gyrodactylus turnbulli* strain
*Gt3*: Laboratory-bred *Gyrodactylus turnbulli* strain
HR: Hazard ratio
IBM: Individual-based model
LA: Lower Aripo River fish
LRT: Likelihood ratio test
MSM: Multi-state model
MKW: Multivariate Kruskal-Wallis test
OS: Ornamental stock
PBM: Population-based model
UKW: Univariate Kruskal–Wallis test
UA: Upper Aripo River fish
