# Supplementary material for: Spatial and temporal parasite dynamics: microhabitat preferences and infection progression of two co-infecting gyrodactylids
Source: Parasit Vectors. 2022 Sep 24;15:336. doi: 10.1186/s13071-022-05471-9 (PMC9508750; doi:10.1186/s13071-022-05471-9)
Supplement: Supplementary file 1 — Additional file 1: Fig. S1. Detailed visualisation of fish heatmaps over eight body regions of fish across parasite strains and fish stocks over time (from day 1 to 17). [file 13071_2022_5471_MOESM1_ESM.pptx]

## Slide 1
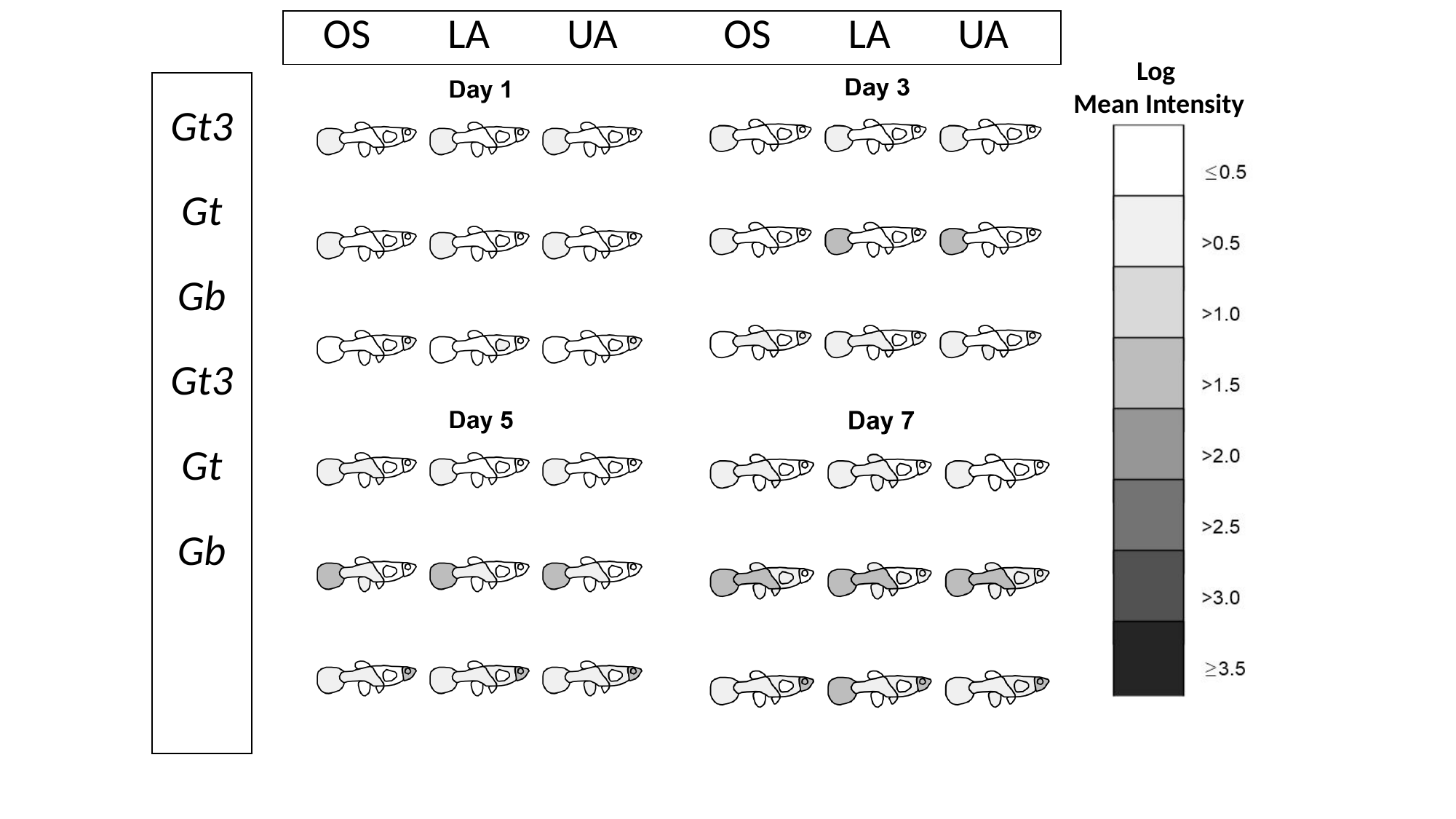

| OS LA UA OS LA UA |
| --- |
Log
Mean Intensity
| Gt3 Gt Gb Gt3 Gt Gb |
| --- |

## Slide 2
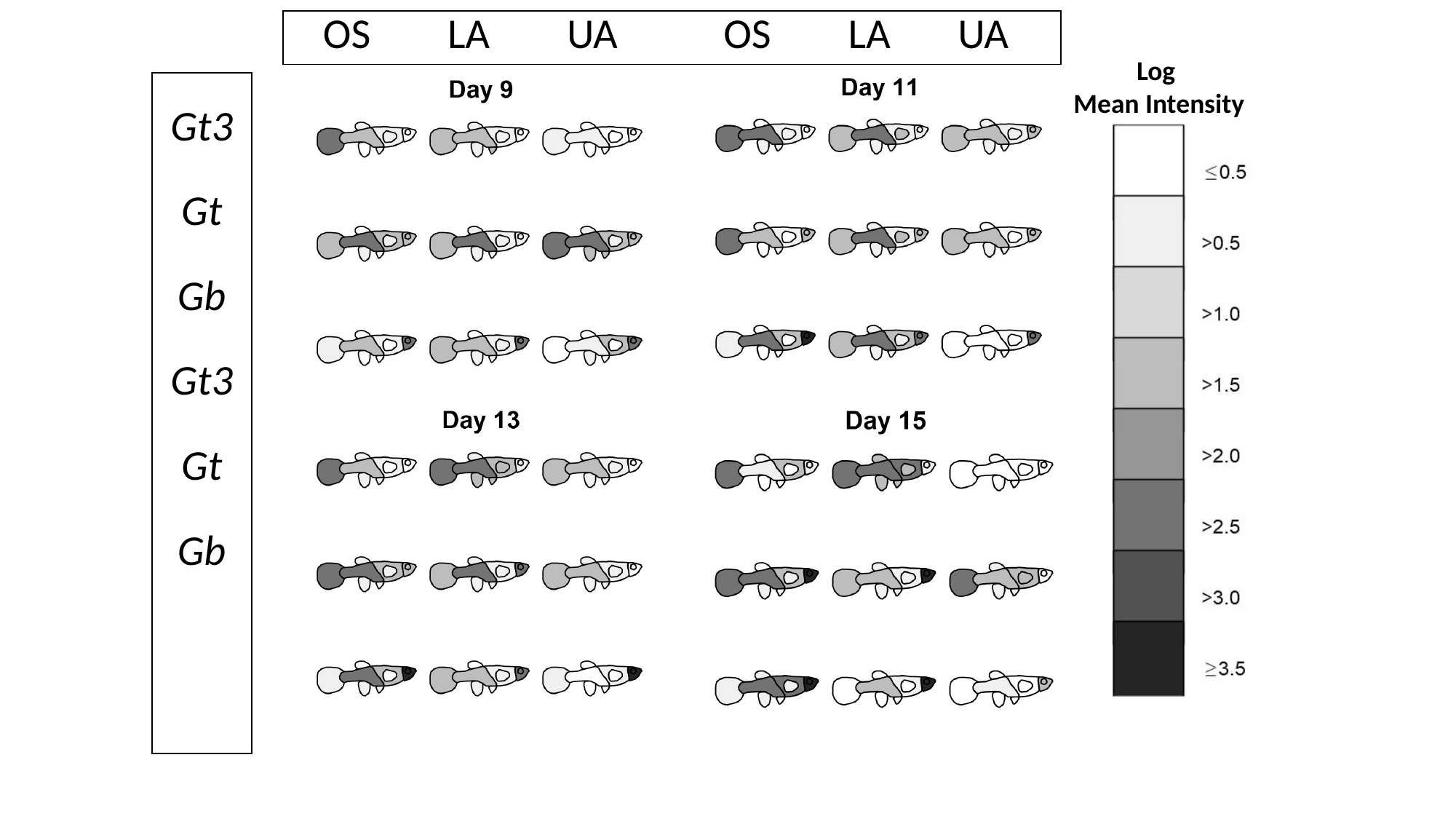

| OS LA UA OS LA UA |
| --- |
Log
Mean Intensity
| Gt3 Gt Gb Gt3 Gt Gb |
| --- |

## Slide 3
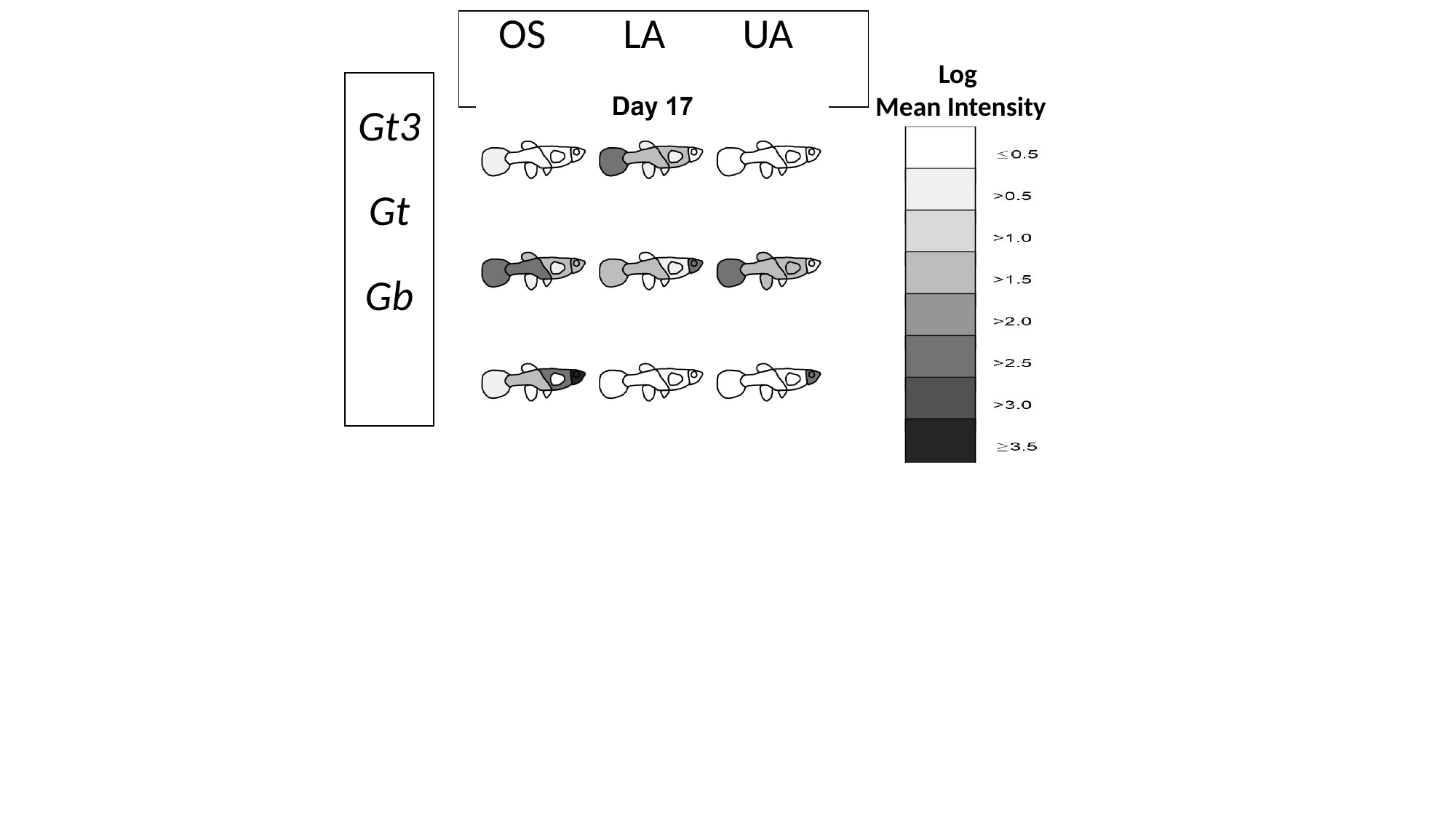

| OS LA UA |
| --- |
Log
Mean Intensity
| Gt3 Gt Gb |
| --- |
