# Supplementary figures and images for: Spatial and temporal parasite dynamics: microhabitat preferences and infection progression of two co-infecting gyrodactylids
Source: Parasit Vectors. 2022 Sep 24;15:336. doi: 10.1186/s13071-022-05471-9 (PMC9508750; doi:10.1186/s13071-022-05471-9)

## Slide 1
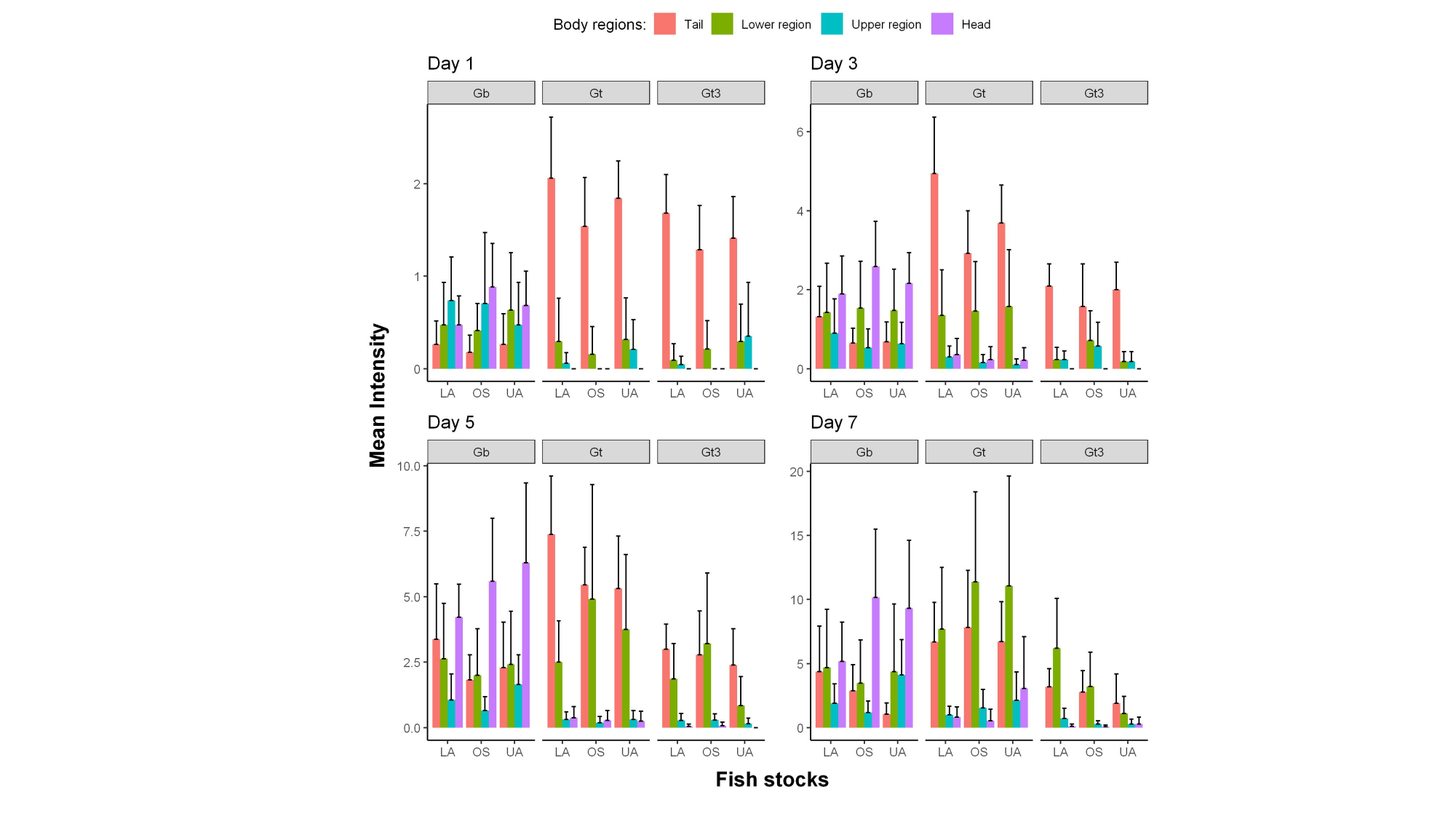

## Slide 2
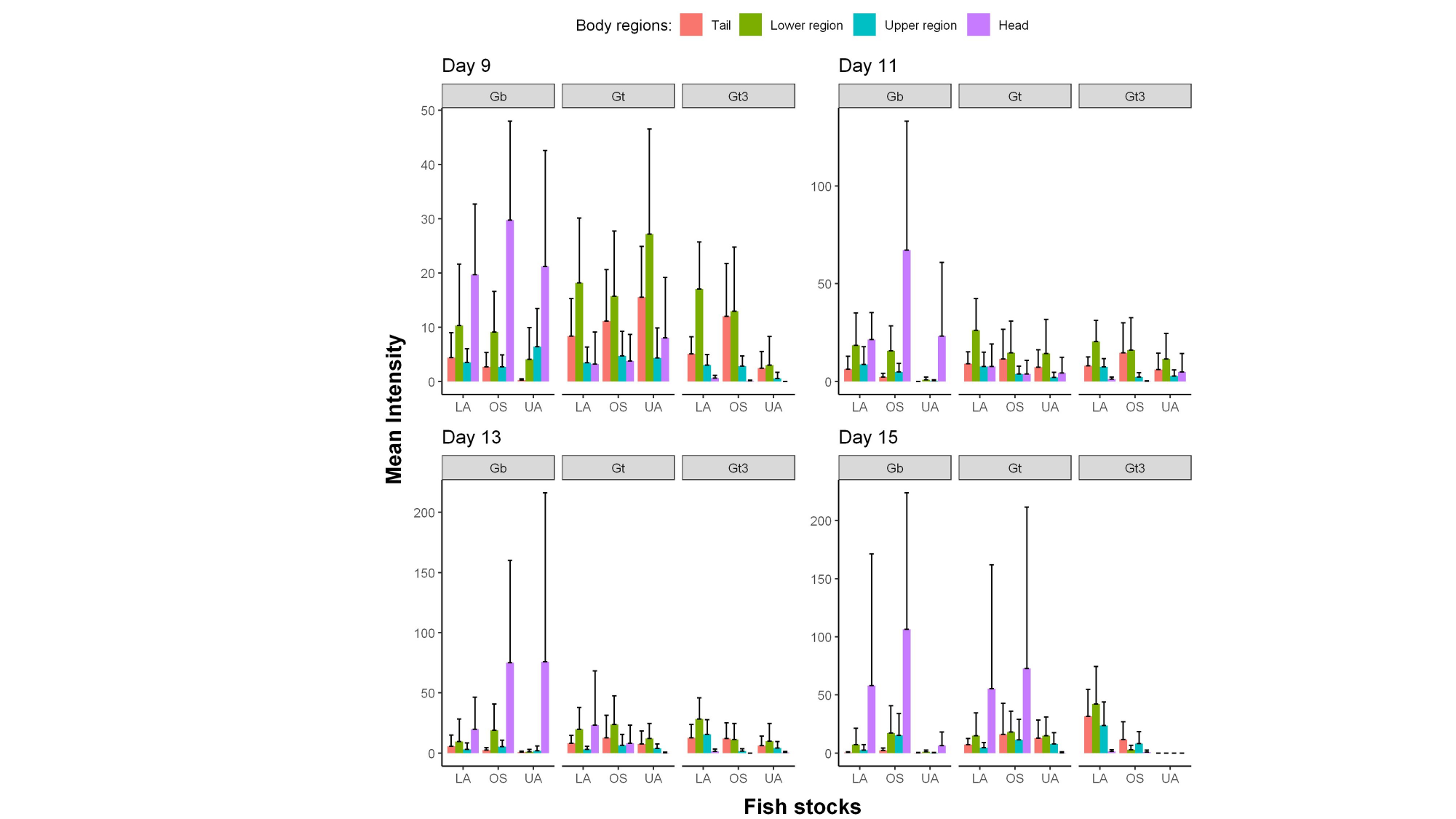

## Slide 3
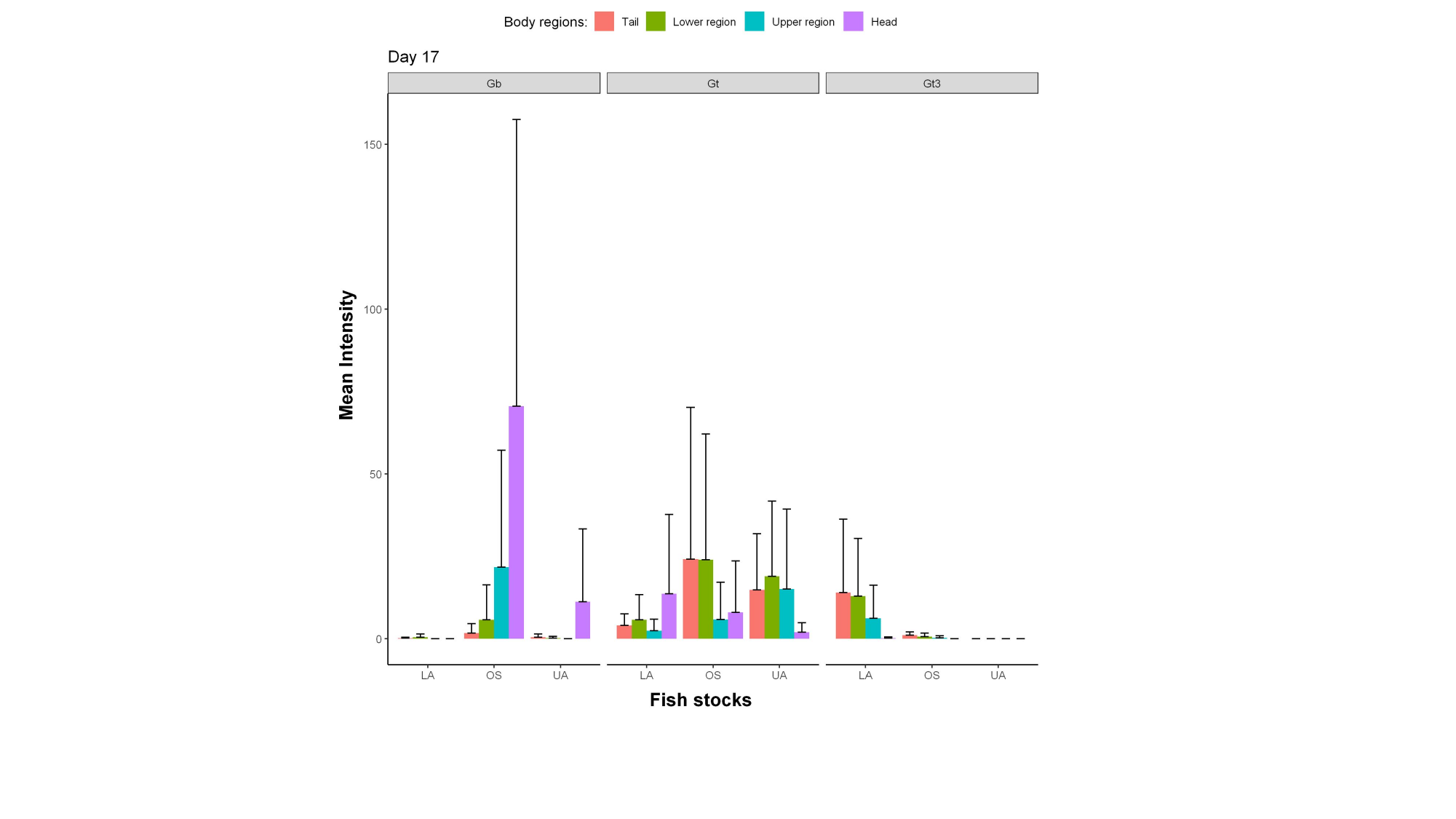

Supplement: Supplementary file 2 — Additional file 2: Fig. S2. Grouped barcharts showing variations in mean intensities at four main body regions of fish across parasite strains and fish stocks over surviving fish and across time (from day 1 to 17). [file 13071_2022_5471_MOESM2_ESM.pptx]
